# Recent progress in nanocomposites of carbon dioxide fixation derived reproducible biomedical polymers

**DOI:** 10.3389/fchem.2022.1035825

**Published:** 2022-10-07

**Authors:** Xin Liu, Zhiwen Jiang, Dejun Xing, Yan Yang, Zhiying Li, Zhiqiang Sun

**Affiliations:** ^1^ Department of Stomatology, China-Japan Union Hospital of Jilin University, Changchun, China; ^2^ Tumor Hospital of Jilin Province, Changchun, China; ^3^ Key Laboratory of Polymer Ecomaterials, Changchun Institute of Applied Chemistry, Chinese Academy of Sciences, Changchun, China

**Keywords:** nanocomposites, carbon dioxide fixation, reproducible, biomedical polymers, decarbonization

## Abstract

In recent years, the environmental problems accompanying the extensive application of biomedical polymer materials produced from fossil fuels have attracted more and more attentions. As many biomedical polymer products are disposable, their life cycle is relatively short. Most of the used or overdue biomedical polymer products need to be burned after destruction, which increases the emission of carbon dioxide (CO_2_). Developing biomedical products based on CO_2_ fixation derived polymers with reproducible sources, and gradually replacing their unsustainable fossil-based counterparts, will promote the recycling of CO_2_ in this field and do good to control the greenhouse effect. Unfortunately, most of the existing polymer materials from renewable raw materials have some property shortages, which make them unable to meet the gradually improved quality and property requirements of biomedical products. In order to overcome these shortages, much time and effort has been dedicated to applying nanotechnology in this field. The present paper reviews recent advances in nanocomposites of CO_2_ fixation derived reproducible polymers for biomedical applications, and several promising strategies for further research directions in this field are highlighted.

## Introduction

With the increases in total population and social productivity, the world’s energy consumption and associated greenhouse gas emissions have increased rapidly (Bauer and Menrad, 2019; Maamoun, 2019; Miyamoto and Takeuchi, 2019; Iglina et al., 2022). Since the industrial revolution in the 1700s, the amount of greenhouse gases emitted into the atmosphere has increased year by year, which has intensified greenhouse effect and caused a series of problems. As the main component of greenhouse gases, CO_2_ released by the combustion of fossil fuels (coal, oil, and natural gas) and products derived from them, plays a leading role in greenhouse effect. In order to avoid the disasters caused by the intensification of greenhouse effect, researchers with different backgrounds have made great efforts in curbing the CO_2_ emissions (Rahman et al., 2017; Pan et al., 2018; Chowdhury and Loganathan, 2019; Xu et al., 2019; Xu et al., 2021).

**FIGURE 1:**
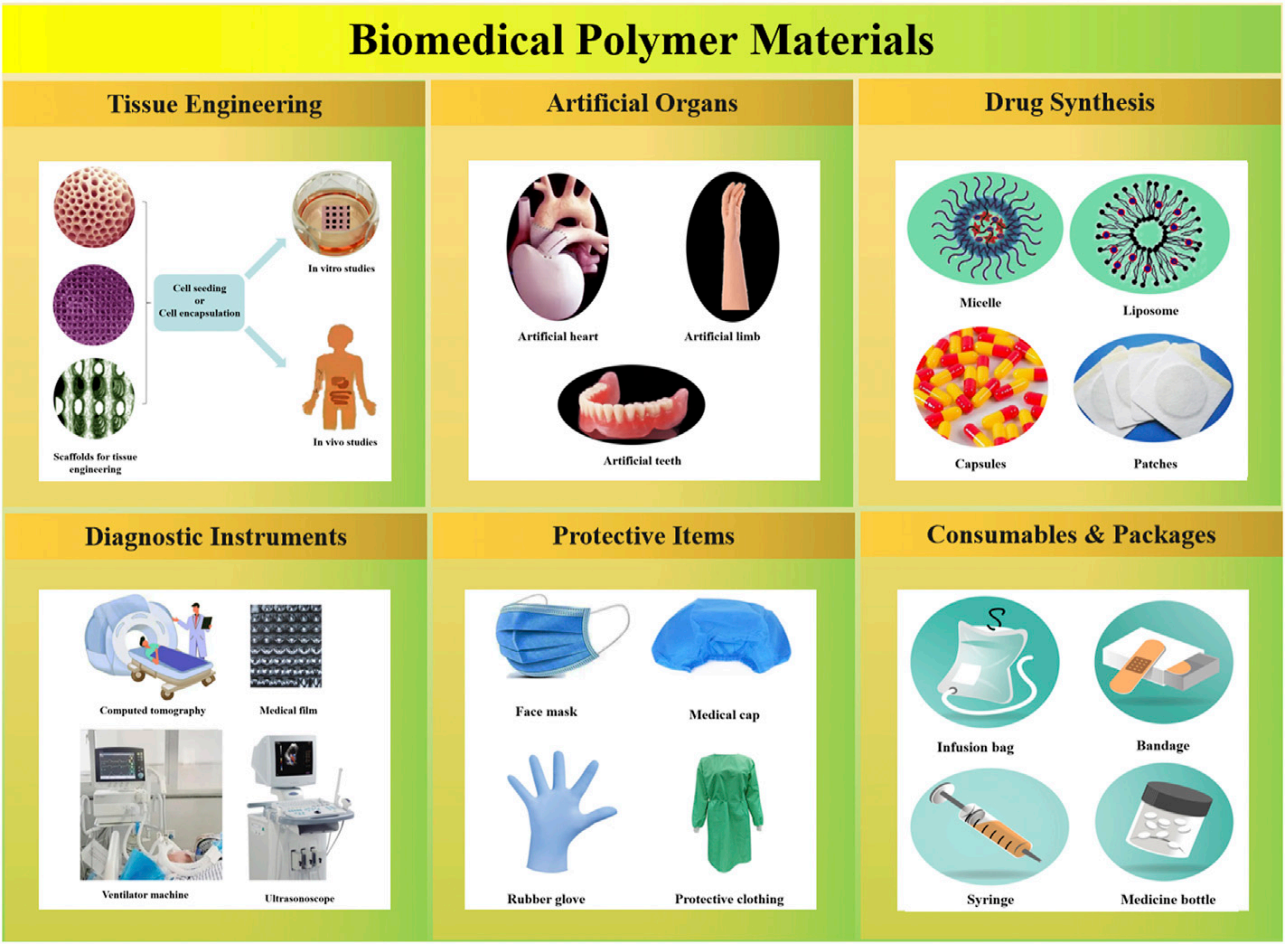
Representative biomedical polymer products applied in different fields.

From the view of front-line medical workers, a lot of biomedical polymer products used in our daily work should share some responsibility for the world’s CO_2_ emissions. Due to their excellent characteristics and ability to reduce the costs, biomedical polymers have been widely used in the fields of tissue engineering, artificial organs, drug synthesis, diagnostic instruments, medical protective items, medical consumables, and packaging materials (Liechty et al., 2010; Mora-Huertas et al., 2010; Tian et al., 2012; Zhang L. et al., 2021; Feng et al., 2021; Hao et al., 2021; Chen W. H. et al., 2022). As shown in Figure 1, except for some large-scale medical equipment, long-life tissue engineering devices and artificial organs, most of the biomedical polymer products have a relatively short life cycle. Most of those used or overdue medical products need to be burned after destruction, which increases the emission of CO_2_. According to the statistics, there are nearly 100 kinds of biomedical polymer materials (Smith and Gambhir, 2017; Shaghaleh et al., 2018; Lyu et al., 2019), and more than 1800 related medical products in the world. From 2011 to 2019, the global production and marketing scale of biomedical polymer materials had increased from 4.391 to 6.771 million tons, with a compound annual growth rate of 5.6%. It means that annual CO_2_ emission by treatment of the biomedical polymer waste will be over 20 million tons. Considering the strict process conditions and high defect rate in the production of medical products, the contribution of greenhouse gas emissions in this field will be even greater. As it is urgent to control the greenhouse effect, the recycling of CO_2_ in biomedical polymer deserves more attention.

**FIGURE 2:**
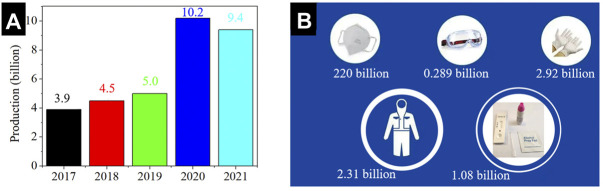
**(A)** Changes in China’s face mask production from 2017 to 2021. **(B)** Epidemic prevention supplies exported from China to other countries in 2020.

Since the outbreak of novel coronavirus disease 2019 (COVID-19), consumption of biomedical materials related to epidemic prevention has soared. As shown in Figure 2A, the production capacity of medical masks in China increased by over 100% after the COVID-19 epidemic. The data released by the Chinese general administration of customs shows that: a large number of medical supplies, including over 220 billion face masks, 2.31 billion protective suits, 289 million pairs of goggles, 2.92 billion pairs of surgical gloves, as well as 1.08 billion COVID-19 virus detection kits were exported abroad from China in 2020 (Figure 2B). Although these epidemic prevention products have played important roles in the global fight against the COVID-19, and have saved countless lives, the environmental problems caused by massive use of them, especially the increase in CO_2_ emissions deserve more attentions.

There are many different ways for classification of biomedical polymer materials (Luckachan and Pillai, 2011; Carrion et al., 2021; Wendels and Avérous, 2021; Chen W. H. et al., 2022; Joseph et al., 2022). According to their degradation behavior, they could be divided into biodegradable and nonbiodegradable biomedical polymer materials (Tian et al., 2012; Pang et al., 2014a; Pang et al., 2014b; Pang et al., 2014c; Sun et al., 2014; Sun et al., 2015b). Based on the ways in which they respond to heat, biomedical polymer materials could be classified as thermoplastics, thermosets and elastomers. Considering their origins, they could also be classified into bio-based and fossil fuel-based biomedical polymer materials (as shown in Table 1) (Endres and Siebert-Raths, 2011; Annabi et al., 2014; Sun et al., 2017; Hacker et al., 2019). In general, the bio-based polymer materials (also known as naturally-derived polymers) produced from reproducible plants, animals, and microorganisms, have less contribution to the greenhouse effect than their fossil fuel-based counterparts (Cui et al., 2011; Jayaramudu et al., 2013; Malwela and Ray, 2015; Jammalamadaka and Tappa, 2018; Sun Y. et al., 2019). As these naturally-derived biomedical polymer materials are fundamentally derived from photosynthesis of plants or other natural CO_2_ fixation reactions, their carbon footprints are much smaller than materials from unsustainable petrochemical raw materials.

**TABLE 1:** Classification of biomedical polymer materials with different origins.

	Origins		Representative varieties^[^**^]^
Biomedical polymer materials	Bio-based	Plants	Cellulose, lignin, starch, alginate, lipids, plant protein (wheat, corn, pea, potato, soy, potato), gums, carrageenan, PLA (typically from starch or sugar cane), etc.)
		Animals	Chitin, chitosan, silk, casein, collagen, whey, hyaluronan, albumin, keratin, leather, etc.
		Microorganisms	PHAs (P_3_HB, P_4_HB, PHBHV, P_3_HBHHx, etc.), PHF, bacterial cellulose, hyaluronan, xanthan, curdlan, pullulan, etc.
	Fossil fuel-based		PE (LDPE, HDPE), PP, PVC, PET, PPT, PU, PGA (mainly from coal), polyamides (PA6, PA66, PA12, PA1212), unsaturated polyesters, PC, PPC, etc.

[**] PLA, poly ([**] PLA, polylactide; PHA, polyhydroxyalkanoate; P_3_HB, poly (3-hydroxybutyrate); P_4_HB, poly (4-hydroxybutyrate); PHBHV, poly (3-hydroxybutyrate-co-3-hydroxyvalerate); P_3_HBHHX, poly (3-hydroxybutyrate-co-3-hydroxyhexanoate); PHF, polyhydroxy fatty acid; PE, polyethylene; LDPE, low density polyethylene; HDPE, high density polyethylene; PP, polypropylene; PVC, poly (vinyl chloride); PET, poly (ethylene terephthalate); PU, polyurethane; PGA, poly (glycolic acid), polyglycolide; PC, polycarbonate; PPC, poly (propylene carbonate).

**FIGURE 3:**
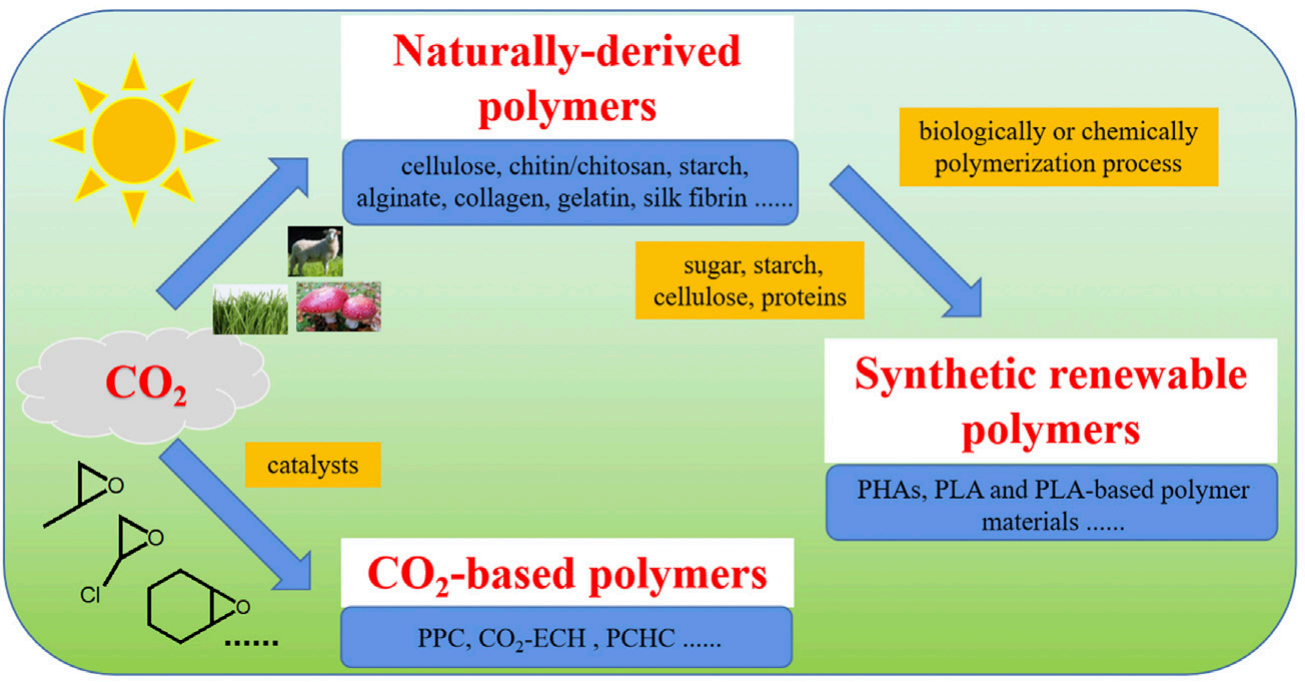
Classification of CO_2_ fixation derived reproducible polymers.

As shown by Figure 3, there are three kinds of CO_2_ fixation derived reproducible polymers, including naturally-derived polymers, synthetic renewable polymers and CO_2_-based polymers. Synthetic renewable polymers are a group of polymeric materials produced by polymerization of monomers derived from raw materials of naturally-based molecules or macromolecules (Hu C. Y. et al., 2018; Duan et al., 2018; Duan et al., 2019; Yang et al., 2019; Zhou et al., 2019; Duan et al., 2021b; Huang et al., 2021). CO_2_-based polymers are produced by polymerization between CO_2_ gas and other bio-based or fossil fuel-based chemicals (Hu C. et al., 2018; Li et al., 2018; Duan et al., 2021a).

As the production of CO_2_ fixation derived reproducible polymers consumes CO_2_, their carbon footprints would be relatively small. Therefore, developing biomedical products based on these CO_2_ fixation derived polymers with reproducible sources, and gradually replacing their unsustainable petrochemical products-based counterparts, will promote the recycling of CO_2_ in this field and reduce the carbon footprints of them. However, compared with the fossil fuel-based biomedical polymers, most of the existing CO_2_ fixation derived polymers from renewable raw materials or CO_2_-based polymer materials, have some shortages in their application properties, which make them unable to meet the gradually improved quality and property requirements of biomedical products.

**FIGURE 4:**
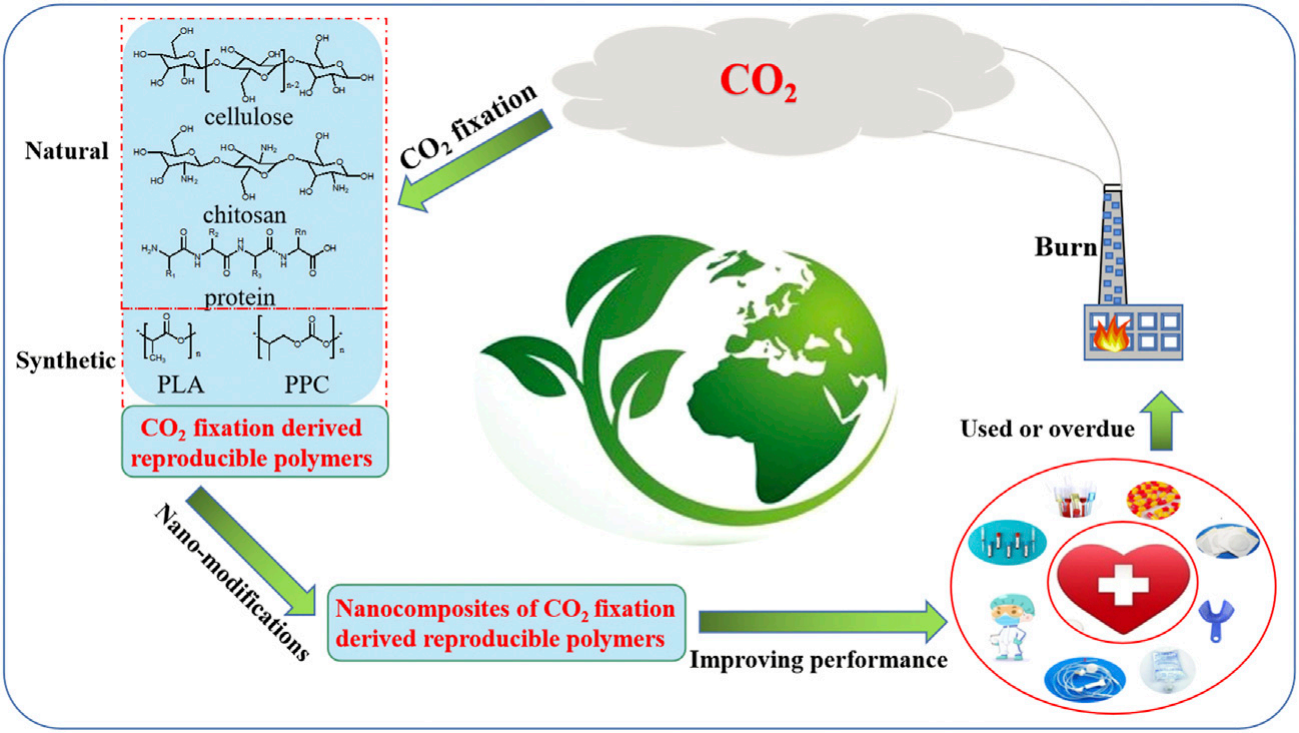
CO_2_ circulation in production and application of CO_2_ fixation derived reproducible biomedical polymers.

In the past few decades, nanotechnology has been proved to be effective in improving the properties of traditional materials (Bosco et al., 2015; Li et al., 2016; Sun et al., 2017; Zheng et al., 2017; Iravani, 2020; Allahyari et al., 2021; Fang et al., 2021; Sajjadi et al., 2021; Tamburaci and Tihminlioglu, 2021; Wang et al., 2021; Iravani, 2022). And many scientists have been engaged in developing new kinds of eco-friendly biomedical polymers by adding various nanomaterials into CO_2_ fixation derived polymers. As shown by Figure 4, natural and synthetic CO_2_ fixation derived polymers could be produced from CO_2_ fixation process. In order to improve their application performance, more and more nanocomposites of these CO_2_ fixation derived reproducible polymers have been prepared by nano-modification. These nanocomposites have wide prospects for production of eco-friendly biomedical polymer products. After being used or overdue, most of the biomedical products produced by nanocomposites of these CO_2_ fixation derived reproducible biomedical polymers would be burnt. The CO_2_ gas released by burning of them could be used to produce new reproducible polymers, so as to realize cyclic utilization of CO_2_. Focusing on the reduction of greenhouse gas emissions in biomedical polymer materials, this short review mainly presented recent advances in the research on nanocomposites of CO_2_ fixation derived biomedical polymers, including naturally-derived polymers (Section 2), synthetic renewable polymers (Section 3) and CO_2_-based polymers (Section 4). Moreover, potential trends, challenges and promising strategies for further research directions in this field are also highlighted.

## Naturally-derived polymers

**FIGURE 5:**
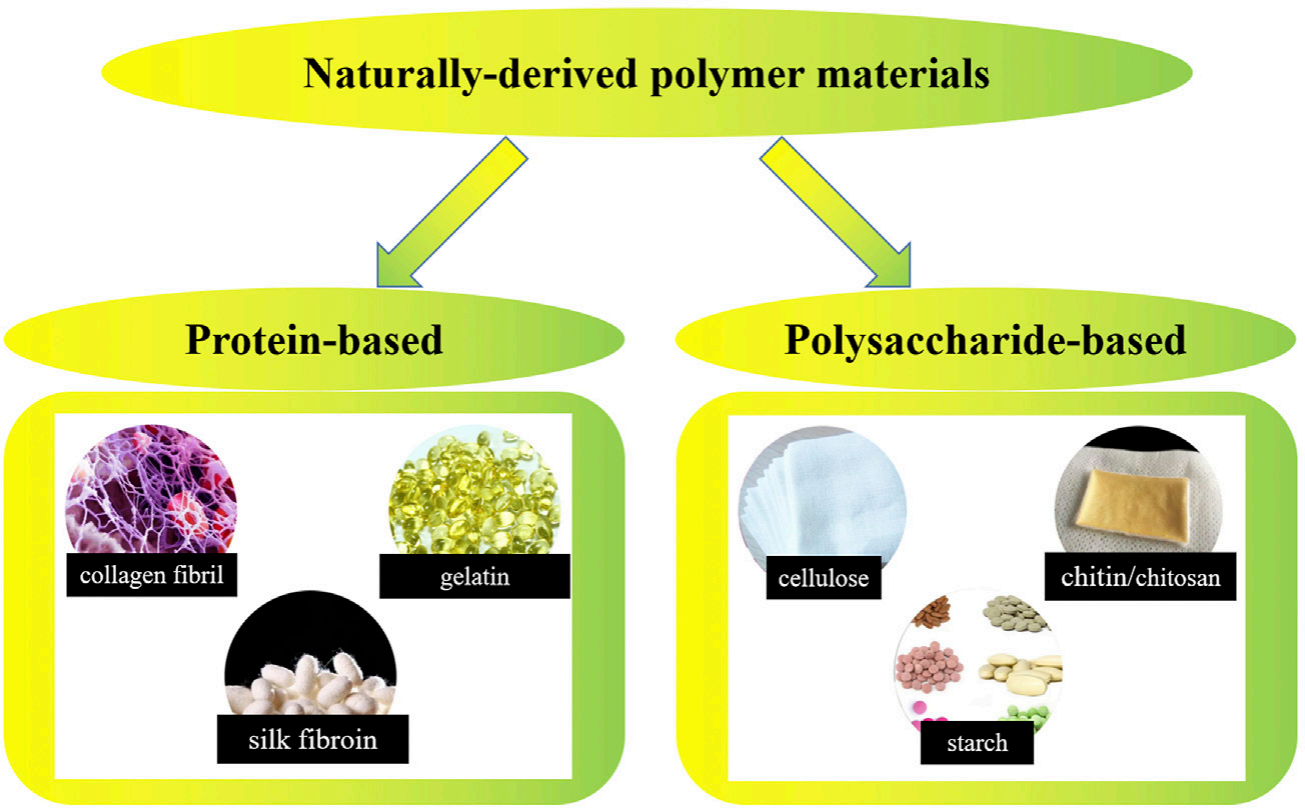
Typical classification of naturally-derived polymer materials.

Naturally-derived polymers generated from living organisms have a long history of being used as biomedical materials. As early as about 3500 BC, the ancient Egyptians began to use cotton fibers or horsehair to suture wounds. Mexican Indians of the same period could use wood chips to repair the injured skull. Since the 1950s, the rapid developed synthetic polymer materials have replaced natural polymer materials in a variety of application fields. However, based on their excellent biocompatibility and biodegradability, naturally-derived polymers still have irreplaceable applications in the field of biomedical materials. Up to now, research on applying naturally-derived polymers in biomedical products preparation is still attractive all over the world (Kokubo and Takadama, 2006; MacDonald et al., 2008; Henkel et al., 2013; Kang et al., 2014; Nguyen et al., 2017; Nurwito and Maulida, 2018; Barroso et al., 2020; Ramanathan et al., 2020; Weller et al., 2020; Neacsu et al., 2021a; Neacsu et al., 2021b; Carrion et al., 2021). As shown by Figure 5, naturally-derived polymer materials could be divided into two groups including protein-based biomaterials (e.g., collagen, gelatin, silk fibrin, etc.) and polysaccharide-based biomaterials (e.g., cellulose, chitin/chitosan, starch, alginate, hyaluronic acid derivatives, etc.). In this section, recent progress in research on nanocomposites of typical naturally-derived polymers and their applications in biomedical products will be presented.

### Collagen

Collagen, the general name of a series of proteins with molecular weight (Mw) ∼300,000, is the most abundant protein in animal body and the major component of skin and skeletal muscle tissues (Chen et al., 2008; Arias et al., 2018; Le Corre-Bordes et al., 2018; Bi et al., 2019; Dippold et al., 2019; Ibrahim et al., 2020; García-Hernández et al., 2021; Hernández-Rangel and Martin-Martinez, 2021). To date, over 20 different types of collagen have been detected in human body, the most common being collagen types I-IV. As most of the collagen molecules have a chain length of sub-micrometer scale, they are a group of natural biomedical nanomaterials. Collagen undergoes enzymatic degradation in the body, resulting in the production of the corresponding amino acids. Due to its unique physicochemical, mechanical, and biological properties, as well as enzymatic degradation, biocompatibility, osteoconductivity, collagen has been extensively researched for biomedical applications (Szentivanyi et al., 2009; Di Maggio et al., 2011; Metwally et al., 2020). Additionally, it has been demonstrated that the rate of collagen deterioration could be controlled by enzymatic pretreatment or by adding cross-links to the polymer chains.

Since collagen and substituted hydroxyapatite are the major solid components of human bone, nanocomposites of collagen and calcium phosphate have been intensively studied as bionic materials for bone replacement and regeneration (Szentivanyi et al., 2009; Henkel et al., 2013). Collagen-hydroxyapatite nanocomposites have gained much recognition as bone grafts, not only due to their composition and structural similarity with natural bone, but also because of their unique functional properties and superior mechanical strength. Incorporating collagen into nanocomposites could provide more cell-recognition sites and result in fast replacement by new bone (Di Maggio et al., 2011; Metwally et al., 2020). Even without osteogenic supplement in culture medium, collagen-hydroxyapatite nanocomposites are very efficient in inducing rapid mineralization by cells.

In addition, collagen-hydroxyapatite based nanocomposites are currently on the market for a wide range of applications. Collagraft^®^, a composite made of fibrous collagen, hydroxyapatite and tricalcium phosphate, has been approved by FDA for using as biodegradable synthetic bone grafts. Floseal^®^, exploiting the high thrombogenic properties of collagen, is a high-viscosity gel hemostatic agent consisting of collagen particles and tropical bovine-derived thrombin. Duragen^®^ suture-free sutures were developed for dural repair and regeneration. Collagen-based wound dressings, like Biobrane^®^ and Promogran^®^, Sulmycin®-Implan gentamicin delivery vehicle, as well as three-dimensional collagen matrix grafts, all show the high research value of collagen in clinical medicine.

### Fibrin

When thrombin transforms fibrinogen, fibrin, a natural polymer involved in the coagulation process would be created. Fibrin is primarily protein with molecular weight ∼360 kDa composed of pairs of polypeptide chains. Its structure consists of a central structural domain composed of fibrin peptide E, two pairs of fibrin peptide A&B molecules, and two terminal structural domains of fibrin peptide D. Based on its good biocompatibility, biodegradability, injectability, and properties to promote cell adhesion and proliferation, fibrin is one of the earliest biopolymers being used as biomedical materials. In recent years, studies on fibrin related to wound healing have been prompted by the fibrous network qualities that naturally generate blood coagulants (Zanetti et al., 2013; Whelan et al., 2014). Currently marketed product Bioseed^®^ is a fibrin-based product for the treatment of chronic wounds made by mixing keratin-forming cells with fibrin. By using autologous plasma, Cryoseal^®^ (Thermogenesis, United States) allows the preparation of hemostatic tissue sealants for the treatment of burn wound care. In addition to the coagulation effect, fibrin networks are used as a sealer on the scaffold surface to improve cell adhesion, proliferation, and encapsulation of multiple cell types in fibrin scaffolds or gels (Yücel et al., 2003). Mittermayr et al. added angiogenesis-stimulating substances to fibrin scaffolds to improve regeneration of ischemic tissue (Mittermayr et al., 2016). Although fibrin with weak mechanical characteristics is susceptible to hydrolysis by hydrolases, its mechanical qualities can be strengthened by combining it with other polymers to create complexes.

### Gelatin

Gelatin is a kind of denatured collagen obtained by hydrolysis of collagen extracted from animal tissues. Therefore, the mechanical properties, swelling behavior and other physicochemical properties of gelatin are determined by the kind of collagen recovered and the technique of transformation. Similar to other naturally-derived polymers, gelatin exhibits excellent biocompatibility, biodegradability and limited immunogenicity. Gelatin has been widely used as a low-cost naturally-derived polymers in medical and pharmaceutical applications (Foox and Zilberman, 2015; Yue et al., 2015; Li et al., 2020; Rajabi et al., 2021). Moreover, many oral capsules and vaccine stabilizers are based on gelatin. Being used as drug delivery vehicles, gelatin could be designed into various carrier systems, including hydrogels, microspheres, nanoparticles, and nanofibers, making it suitable for drug delivery to different organs. Gelatin nanoparticles, for instance, are more suitable for drug delivery applications within the brain (Xavier Mendes et al., 2021). Nanocomposites of gelatin blended with other complexes are currently under numerous investigations for improving the potential applications in tissue engineering. For example, Elsayed et al. used the fabricated bionic electrospun gelatin fiber scaffold for the mid-membrane equivalent of tissue engineered vascular grafts, and the scaffold exhibited good smooth muscle cell proliferation capabilities (Elsayed et al., 2016).

### Chitin/chitosan

Composed of renewable, biodegradable carbohydrates, chitin and chitosan are the second abundant biopolymers in nature (Niaounakis, 2014; Fernandes et al., 2022). Based on their excellent cell attachment and growth abilities, chitin and chitosan made up of N-acetyl glucosamine and N-aminoglucose monomers, have been widely applied in biomedicine (scaffolds, gels, particles, films, etc.) (Qin et al., 2018; Wu et al., 2018; Xu et al., 2018; Yu et al., 2018). The positive charges of chitosan enhanced its capacity to adsorb negatively charged cells. Being used as wound dressing material, chitosan exhibits outstanding hemostatic and antimicrobial properties. Cui et al. (2011) found that chitosan-collagen hydrogels containing staphylococcin not only could promote the burn wound healing, but also showed antimicrobial efficacy against methicillin-resistant *Staphylococcus* *aureus* . Based on the hemostatic function of chitosan, products such as HemCon^®^ bandages (HemConMedical Technologies, Inc.) and Celox gauze (MedTrade) have been created and commercialized for the treatment of localized wound bleeding (Bennett, 2017). Chitosan has also been widely used in the field of tissue engineering scaffolds. Its regenerative capacity as a scaffold can be further enhanced by loading the structure with some bioactive compounds. It has been found that filamentous protein/chitosan-based scaffolds, used for repairing myofascial defects in the abdominal wall, showed sustained integration and similar mechanical strength with adjacent natural tissues (Gobin et al., 2006). Colloidal particles formed by chitosan could encapsulate a variety of biomedical molecules, which give them excellent potential in applications as drug delivery systems. Khangtragool’s experiments had successfully used chitosan as drug delivery vehicle for vancomycin application in the rabbit body eye. However, chitosan has mechanical weaknesses and needs to be used in combination with other materials for bone tissue engineering applications (Sharifianjazi et al., 2022). Isikli et al. demonstrated that: compared to pure chitosan scaffolds, nanocomposite scaffolds of hydroxyapatite/chitosan had improved Young’s modulus and compressive strength, while promoted cell attachment and value-added capacity of the scaffold (Isikli et al., 2012). Tamburaci et al. created composite scaffolds for the regeneration of bone tissue by adding diatomaceous earth into chitosan membranes (Iravani, 2020). This kind of diatomaceous earth-doped chitosan composite membranes were proved to have increased swelling behavior, protein adsorption capacity and surface crudity. Moreover, the composite membranes showed excellent biocompatibility by boosting the proliferation of Saos-2 cells and alkaline phosphatase activity.

### Starch

Starch is one of the major carbohydrates commonly present in plant tubers and seed endosperm, such as potato, maize and wheat, etc., (Duan et al., 2011; Ismail et al., 2013). Taking advantage of the low price, easy availability, biocompatibility and biodegradability, starch nanocrystals have been widely used in biomedical field. In tissue engineering applications, adding starch to hydroxyapatite could not only enhance the material’s capacity for osteointegration, but also improve its thermal-mechanical properties and cell adhesion behaviors. Mahdieh et al. (2016) created a kind of novel nanocomposite by combining thermoplastic starch and poly (vinyl alcohol) (PVA) with nanoscale magnesium olivine and vitamin E, respectively. Compared with standard starch-PVA matrix, the novel nanocomposite showed better biological and mechanical properties, lower rate of degradation and increased secondary osteoblast proliferation. Moreover, chemically modified starch could be used for drug delivery. The nonionic nature of starch makes it easy to co-blend with other polymers, thereby increasing the pore size and water absorption capacity of the co-blended polymer (Ngoenkam et al., 2010). de Oliveira Cardoso et al. (2017) prepared hydrogels of gellan gum and starch retrograded blends with or without ketoprofen. By changing the concentrations of polymer and cross-linkers, properties of the starch hydrogels could be controlled. The hydrogels with adjustable mechanical and structural properties were promising materials for drug delivery of different therapeutic needs (Ghavimi et al., 2015). In order to overcome the limited shear and heat resistance of starch, SPCL has been prepared by blending starch with the biodegradable polycaprolactone (PCL). Addition of PCL could reduce the stiffness and high water-sensitivity of starch, and made improvement of SPCL in processability, degradability, mechanical properties and cell proliferation (Labet et al., 2007). Additionally, addition of starch could enhance the biodegradability of PCL, and significantly reduce the production cost of the final product (Morgan et al., 2013).

### Cellulose

As the most abundant natural polymer on earth, cellulose is considered as the most important renewable biological resource, with great economic value in the world (Neo and Yang, 2015; Basu et al., 2018). Cellulose is a linear polymer composed of β-D-glucopyranose units (disaccharides) linked by glycosidic β-(1,4) bonds. Nanofibrillar networks formed by plant cellulose have a thickness of about 20–100 nm. Whereas bacterial cellulose nanofibrils are 20–50 nm thick. Bacterial cellulose usually shows lower crystallinity, and is better at storing water than plant cellulose (Lavoine et al., 2012; Olson et al., 2012). Additionally, the plant cellulose’s glycan polymers are resistant to biotransformation, which restricts its application fields (Loh et al., 2018; Mishra et al., 2018). Therefore, bacterial cellulose is the subject of extensive investigation currently. Since the 1980s, bacterial cellulose has been used as a natural polymer for wound dressing. Fu et al. (2013) applied a mixture of bacterial cellulose hydrogel and acrylic acid solution in a mouse allograft wound model, and the bacterial cellulose hydrogel exhibited good cell carrier properties and accomplished allograft wound healing in the model. Nanofibrillar products such as Biofill^®^ (Fibrocel, Brazil) or Nanocell^®^ (Thai Nano Cellulose Co. Ltd., Thailand) based on bacterial cellulose are widely used in clinical settings for various types of wounds, such as chronic ulcers, donor partial adjuvants, and wounds following skin cancer (Muangman et al., 2011; Islam et al., 2021). In addition, bacterial cellulose also could be used in medicine delivery systems. Silver nanoparticle-based bacterial cellulose (Ag-BC) has antibacterial effect against both Gram-positive and Gram-negative microorganisms (Yadav et al., 2015). In the domains of orthopedics, otology, ophthalmology, urology, and neurosurgery (Meyer and Palmer, 1934; Bodin et al., 2010; Wang et al., 2010; Kowalska-Ludwicka et al., 2013; Martínez Ávila et al., 2014). Bacterial cellulose is widely applied as a scaffold for guided tissue regeneration. In the realm of tissue engineering, it has an indispensable status.

### Hyaluronic acid

Hyaluronic acid (HA) was first isolated from the vitreous fluid of the eye by Meyer and Palmer in 1934 (Monteiro et al., 2014). As an anionic linear polymer composed of D-glucuronic acid and N-acetyl-D-glucosamine, HA could be found in all living organisms. Similar to other natural polymers, HA’s biocompatibility and biodegradability make it one of the most widely used carbohydrate-based natural polymers in the field of tissue engineering. In the past two decades, HA has been involved in a wide range of biological activities, including the control of cell motility and adhesion, promotion of wound healing, and drug delivery in tissue engineering scaffolds. HA binded to CD44 and HA-mediated motor receptor (RHAMM) could be used as cell surface receptors. Internalization triggered by CD44-HA binding can prevent CD44 overexpression in cancer cells, making it possible to distribute anticancer drugs (Simman et al., 2018). Numerous biomedical materials based on HA derivatives have been commercialized, including esterified derivatives like ethyl/benzyl esters (HYAFF^®^) and cross-linked HA gels that have been extensively investigated for use as wound dressings. Mucoadhesive HA solutions (SYNVISC^®^ and ORTHOVISC^®^) are also used in clinical settings as synovial fluid substitutes for the purpose of reducing pain and improving joint mobility in patients with osteoarthritis. The AMVISC^®^ vitreous fluid replacement for ophthalmic applications is based on HA, which protects sensitive eye tissue during eye surgery. In the field of tissue regeneration, the HA-based scaffold Hyalomatrix^®^ (Anika therapy) can be employed as a dermal substitute for severe surgical wounds, which can promote capillary growth and attract fibroblasts to grow inward after delivery to the wound bed (Faga et al., 2013; Dwivedi et al., 2020).

### Alginate

Alginate, also known as sodium alginate, is an anionic polysaccharide found in the cell wall and intercellular space of algae. It is a block copolymer composed of two different ratios and arrangements of glycuronates ((1,4)-linked β-d-mannuronic acid and α-l-guluronic acid), which allows aqueous solutions of alginate to exhibit a non-Newtonian behavior similar to glycosaminoglycans. Due to its excellent properties including non-toxicity, biocompatibility, non-immunogenicity, straightforward gelation, controlled degradability and cheap in price, alginate has been used in a wide range of biomedical applications. Alginate gels could encapsulate molecules or cells because of their gentle characteristics (Lim and Sun, 1980; Gasperini et al., 2014). In the 1980s, alginate containing encapsulated pancreatic cells was first implanted into humans (O’Meara et al., 2015). Commercial products based on alginate derivatives, such as Kaltostat^®^ and AlgiSite^®^, are frequently used in wound care, mainly for the treatment of venous leg ulcers, donor wound dressings, pressure ulcers, and so on (Lee and Mooney, 2012; Dumville et al., 2014; Brenner et al., 2015; Khoshzaban et al., 2018; Langa et al., 2018). Alginate hydrogels can also be used as scaffolds and matrices for the capture and delivery of bioactive molecules or cells, and are widely used for tissue regeneration. Alginate can also be used as a drug delivery system, and encapsulation of particular proteins and bioactive substances in alginate gels can enhance their targeting.

## Synthetic renewable polymers

Synthetic renewable polymers are a group of polymeric materials produced by biologically or chemically polymerization process (Modjarrad and Ebnesajjad, 2013; Takeuchi et al., 2013; Sun et al., 2015a; Duan et al., 2015; Sun et al., 2016; Duan et al., 2017a; Duan et al., 2017b; Ricklefs et al., 2017; Yang et al., 2022). The production of monomers for this kind of polymer consumes naturally-based molecules or macromolecules (e.g., sugar, starch, cellulose, proteins, etc.) as raw materials. Originated from photosynthesis CO_2_ fixation process of plant or microorganisms, their carbon footprints are lower than traditional fossil fuel-based polymers. The premise of discussing the impact of a material on greenhouse effect is that its production and application should reach a certain scale. So far, the most intensively studied synthetic renewable polymers, which are expected to make significant contributions to decarbonization, namely polyhydroxyalkanoates (PHAs) and polylactic acid (PLA) related polymer materials.

### Poly (hydroxyalkanoates)

**FIGURE 6:**
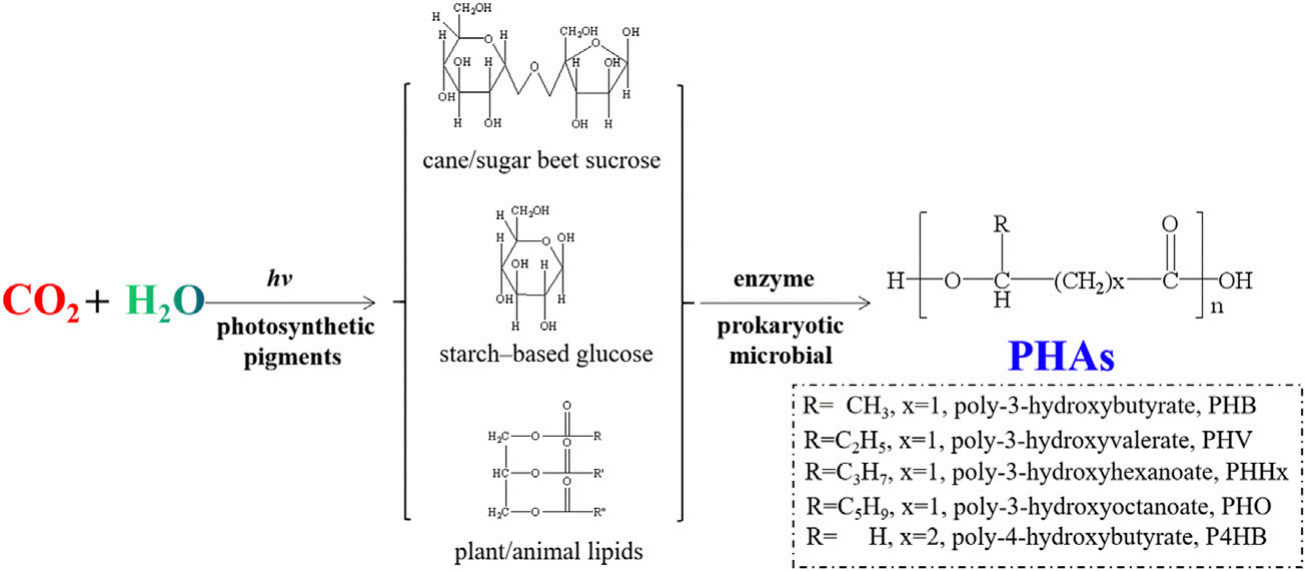
Schematic illustration for synthetic route of PHAs with different monomeric building blocks.

In the past few decades, as a group of intracellular polyesters synthesized by different microorganisms, PHAs have been developed rapidly as a kind of new biosynthetic renewable polymers. PHAs are the largest group of biopolymers, and new types of them can be manufactured by biosynthesis with genetically modified bacteria. As shown by Figure 6, based on the feed stocks such as cane/sugar beet sucrose, corn starch-based glucose, plant oils (soybean oil, palm oil, corn oil, etc.) and animal lipids, PHAs with different monomeric building blocks could be produced by biosynthesis process catalyzed by enzymes in living cells. The easy availability, biocompatibility and biodegradability of PHAs make them suitable for biomedical applications, such as absorbable sutures, drug delivery systems, scaffolds and implants to support stem cell proliferation materials (Pourmollaabbassi et al., 2016). Lim et al. compared the properties of different PHA scaffolds in the field of bone tissue engineering, showing well bioactivity and the ability to induce bone regeneration (Lim and Sun, 1980). Bagdadi’s experiments proved the excellent performance of poly-3-hydroxyoctanoate in cardiac tissue engineering (Bagdadi et al., 2018). In addition, PHAs can be applied as wound dressings for skin defect repair. Shishatskaya et al. (2016) employed poly (3-hydroxybutyrate- co-4-hydroxybutyrate) solutions as experimental wound dressings and discovered that they promoted wound tissues healing and vascularization. It has also been found that PHA alone can stimulate cell cycle progression, reduce mitochondrial reactive oxygen species (ROS) production, decrease neuronal mortality, and inhibit neuronal membrane potential changes for the treatment of neurodegenerative illness and seizures control (Yum et al., 2012). Due to its high degradation rate, PHAs could be utilized as a substitute for disposable plastics such as medical appliances, medical packaging and medical trash-bags (Lee and Mooney, 2012; Lim et al., 2017). Although the market prices of these products are not high, the total consumption of them is huge, which are of great significance for decarbonation and environmental protection.

### Polylactic acid

**FIGURE 7:**
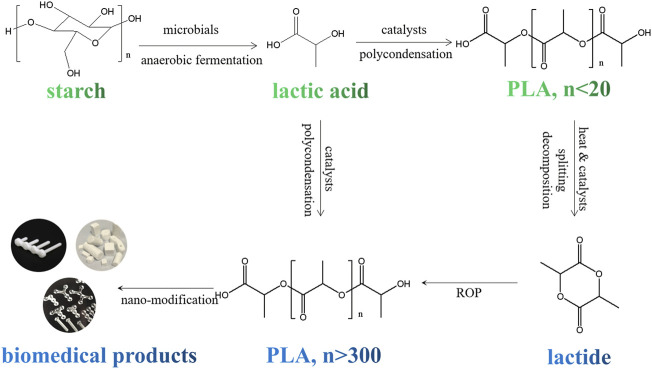
Schematic illstration for preparation process of PLA based biomedical products from renewable raw materials.

PLA is one of the most widely utilized synthetic renewable polymers in the biomedical field. As a hydrolyzable aliphatic semi-crystalline polyester, PLA can be prepared both by direct polymerization of lactic acid and by ring opening polymerization (ROP) of lactide (Figure 7) (Langa et al., 2018). The monomers of PLA could be generated by fermentation of carbohydrates (maltose, sucrose, lactose, etc.), which makes it renewable and sustainable. PLA with different stereoisomeric structures show distinct properties (Abd Razak et al., 2012; Khemani and Scholz, 2012; Pang et al., 2014b; Pang et al., 2014c; Sun et al., 2014; Sun et al., 2015b; Rokutanda et al., 2015; Hadasha and Bezuidenhout, 2017; Zhang et al., 2017). For example, poly (D-lactic acid) (PDLA) and poly (l-lactic acid) (PLLA) are semi-crystalline materials, and poly (D, l-lactic acid) (PDLLA) and meso-PLA are amorphous materials (Giménez et al., 2018).

Despite its complicated molecular stereo structure, PLA shows many advantages in terms of chemical and mechanical properties, biocompatibility and biodegradability. A variety of PLA-based biomedical products have been developed, including surgical clips for surgical instruments, sutures, adhesives for wound closure, bone staples and plates, regenerative scaffolds and drug delivery systems, even medical devices and packaging, etc. Among them, PDLLA is widely used as biomedical coating for orthopedic materials because of its excellent mechanical stability and biocompatibility. Low molecular weight PDLLA can be bonded to antibiotics and other drugs for local drug delivery. Because of its nontoxicity, biocompatibility, and biodegradability, PLLA has been widely explored for cardiac tissues.

Gimenez’s experiments used PLLA scaffolds with overexpression of connexin43 (CX43) to line diaphragm myoblasts (DM) in a model of acute coronary artery occlusion in sheep. The results showed that PLLA plate inoculated with DM overexpressing CX43 could reduce the infarct size, decrease the fibrosis degree of infarct boundary, and induce cardiovascular regeneration, so as to improve cardiac function (Arya et al., 2018). PLLA has relatively high tensile strength, low elongation, and superior modulus (∼4.8 GPa). And it takes time from months to years to for PLLA derided products to lose mechanical integrity. These properties make PLA more suitable for load-bearing roles (e.g. orthopedic fixation). Numerous PLA-based orthopedic products are available on the market, such as Bio-Anchor^®^ (Conmed), Maniscal Stinger ^®^ (Linvatec) and Phantom Suture Anchor^®^ (DePuy). The ability of PLLA to form high-strength fibers can also be used to develop absorbable row sutures and scaffold materials to replace non-degradable fibers. Meanwhile, PLLA has applications in tissue engineering, for example, Sculptra^®^, an injectable product of PLLA, helps HIV patients who are losing face fat.

### PLA related polymers

In the field of biomedical applications, poly (lactic-co-glycolic acid) (PLGA), copolymer of PLA and poly (glycolic acid) (PGA), is considered to be the most representative PLA related polymer materials (Mulinti et al., 2018). The degradation and mechanical properties of PLGA can be adjusted by varying the ratio of lactic acid (or lactide) and glycolic acid (or glycolide). With excellent properties of biocompatibility, non-cytotoxicity and processability, PLGA has been widely used for biomedical and tissue engineering applications (Lanao et al., 2013). For example, PLGA can be utilized as various drug delivery vehicles (microspheres, nanospheres or nanofibers) for the controlled release of drugs, such as for the treatment of cancer and the release of hormone-based drugs. Zamani et al. (2015) developed a novel SDF-1α delivery system by using coaxial electrospraying. The SDF-1α incorporated PLGA particles they achieved had distinct core-shell structure, with the shell thickness of ∼300 nm. Their experiment results showed that the as-prepared core-shell particles could realize controlled release of the SDF-1α for 40 days Moreover, as one of the potential candidates for application in tissue engineering, a large number of researchers have applied PLGA in scaffold development studies. Liu et al. (2012) showed that nanocomposite material of silver nanoparticle/PLGA stainless steel alloy had strong antibacterial and osteoinductive properties, indicating its potential for bone regeneration applications. Lin et al. (2019) applied a tyramine-modified bilayer PLGA scaffold in a porcine model and found that chondrocytes in the scaffold with small and large pores in the upper and lower parts, respectively, had the ability to promote cartilage regeneration and articular cartilage repair. So far, there are many marketable products based on PLGA. For example, Vicryl^®^, the most popular clinically used absorbable suture, is prepared by PLGA copolymer with 90% glycolic acid and 10% l-lactic acid. Vicryl Rapid^®^, the irradiated version of Vicryl^®^, shows to be quick in degradation. By increasing the l-lactic acid/glycolic acid ratio in PLGA, ANACRYL^®^ provides absorbable sutures with slow degradation rate. In all, depending on the application site, sutures with varying degradation rates can be selected to achieve the best healing effect. Tissue-engineered skin grafts (Dermagraft^®^) are the products marketed based on PLGA polymers for use as skin replacement materials.

Due to its biodegradability, the use of PLA related polymer materials in packaging materials and other disposable products has already received significant attentions (Sinclair, 1996; Haugaard et al., 2002; Frederiksen et al., 2003). Recently, more and more research works related to development of PLA-based nanocomposites for applications in biomedical devices, packages, and consumable products have been reported. Nonato et al. reported the preparation of PLA nanocomposites containing ZnO nanofibers by solvent-cast 3D printing method. This kind of PLA/ZnO nanocomposites were expected to be applicable in medical packaging applications (Nonato et al., 2019). Using hydroxyapatite with micro- and nano-particles as filler, Zaharescu et al. studied the effect of particle size on stability of PLA-based composites. It had been demonstrated that composites with nanosized filler showed better stability under gamma-irradiation. The results of this work would do good to developing long shelf-life PLA-based products for medical wears, face masks and other disposible devices (Zaharescu et al., 2020). Focusing on developing new materials for food and drug containers, packaging items, medical devices, Aversa et al. studied the wear resistance property of injection moulded PLA-talc composites detailedly. By constructing PLA super-networks of oriented and pyknotic crystals with the assistance of ductile poly (butylene adipate-co-terephthalate) (PBAT) nano-fibrils, Zhou et al. prepared PLA composite film with excellent gas barrier performance, high strength, and toughness, which showed promising prospect to be applied in the field of pharmaceutical packaging (Zhou et al., 2016). Antimicrobial and photodegradation properties of PLA/CaO nanocomposites were studied by Loyo et al. (2022). Results of their experiments showed that incorporating CaO nanoparticles into the PLA could be an applicable strategy for development of eco-friendly new medical packaging and devices. Zhao et al. (2019) studied effect of sterilization methods on commercially available biodegradable polyesters, focusing on their potential applications for single-used medical products. Their experiments demonstrates that, by choosing a suitable sterilization process, PLA could have potential to be used for production of transparent medical devices (the barrel of syringes or microfluidic chips), while PLA/PBAT blends might be applied in preparing non-transparent medical packaging.

## CO_2_-based polymers

**FIGURE 8:**
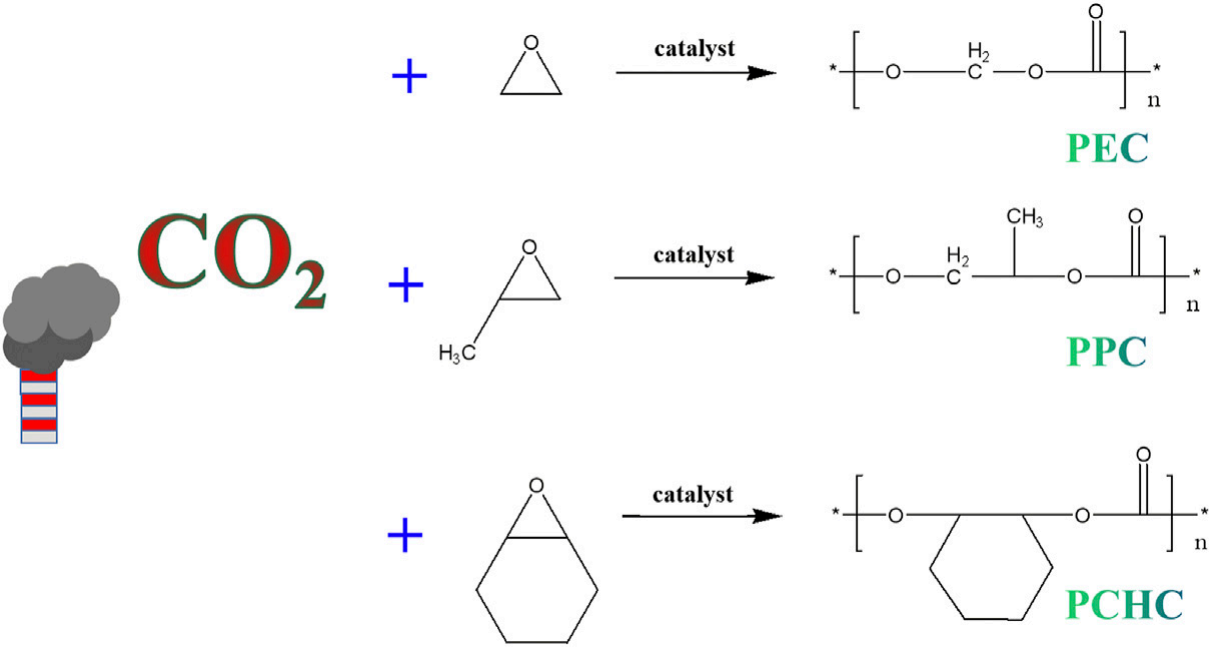
Synthetic routes for typical CO_2_-based polymers prepared by ROCP of CO_2_ and epoxides.

Among the various strategies for CO_2_ fixation, to produce CO_2_-based polymers offers significant potential (Hu C. et al., 2018; Li et al., 2018; Duan et al., 2021a). The CO_2_-based polymers usually are prepared by ring-opening copolymerization (ROCP) of CO_2_ and epoxides (Figure 8), under the assistance of certain catalysts (Li et al., 2018). Most of the CO_2_-based polymers produced by this way are aliphatic polycarbonates, which could be used for replacing the petroleum plastics in some applications such food and medical packaging, agricultural films, trash bags, etc. Moreover, low molecular weights CO_2_-based polymers could be used to synthesis poly (ether carbonate) polyols. Replacing petrochemical polyether polyols with these CO_2_-based polymers derived poly (ether carbonate) polyols in polyurethane manufacturing would lead to significant reduction in greenhouse gas emissions (∼20%). Currently, the annual requirement for polyols in field of for polyurethanes production is ∼3–4 Mt (Hemamalini and Dev, 2018; Jafari et al., 2018; Bauer and Menrad, 2019; Gibb, 2019; Shokraei et al., 2019; Heath et al., 2020). Therefore, the replacement of polyether polyols by poly (ether carbonate) polyols of CO_2_-based polymers could add value to waste CO_2_ emissions, and do good to decarbonization.

### Poly (propylene carbonate)

PPC is an amorphous aliphatic polycarbonate produced by alternating copolymerization of carbon dioxide and propylene oxide (Fu et al., 2000; Liu et al., 2015). The low price of propylene oxide and CO_2_ has made PPC one of the most extensively researched and promising eco-friendly synthetic polymers. Since PPC is non-toxic, non-polluting, highly transparent, renewable and degradable, barrier, malleable and superior electrical conductivity, it can be used for preparation of various biomedical products (Dånmark et al., 2011; Weems et al., 2021). However, with side chains of -CH3 on its main molecular chain, PPC has drawbacks of poor dimensional stability and a low glass transition temperature (Tg), which restrict its usage in high-temperature resistance application fields. On the other hand, the degradation products of PPC *in vivo* are water and carbon dioxide, which can avoid the adverse effects similar to that produced by PLA and PLGA and have less inflammatory reactions. This facilitates the develop of PPC as a scaffold for tissue engineering applications (Liu et al., 2015). Manavitehrani et al. developed a gas foam porous scaffold designed based on PPC, starch and bio-glass. Both *in vitro* and *in vivo* experiments showed well biocompatibility and tissue infiltration. And in a joint implantation model, bone tissue integration was observed (Manavitehrani et al., 2019). In another experiment, PLA, tricalcium phosphate (TCP) nanoparticles and PPC were mixed to form a novel polymer scaffold named for PPTE for short. By inoculating mouse osteoblasts onto the PPTE composite, the researchers had found that the PPTE composite could promote attachment, proliferation, and mineralization of osteoblast. Meanwhile, PPTE composites also could control the production of bone bridge proteins, and promote the activity of phosphatase, making them viable materials for applications in bone regeneration (Fang et al., 2015). Compared to conventional degradable biopolymers, PPC exhibits better flexibility and maneuverability, making it an ideal carrier for drug delivery and a polymeric drug retardation material (Li et al., 2004; Manavitehrani et al., 2019). Using sirolimus-eluting PPC mesh in a rat jugular vein to abdominal aorta autograft model, it showed that PPC mesh could decrease arteriovenous graft stenosis and suppress MMP-2 and MMP-9 expression (Sun H. et al., 2019). In a recent study, single/double epoxy-capped PPC and chitosan were combined to form chitosan/PPC nanoparticles (CS/PPC NPs). The polymer was then tested for chemical and antibacterial properties. Experiment results revealed that the CS/PPC NPs had a high level of antibacterial activity against *Escherichia coli* and *Staphylococcus aureus*, providing a novel strategy for the manufacture of novel nano-size antibiotics (Quan et al., 2021).

Because of its good water barrier and low gas permeability, PPC shows strong and long-lasting antibacterial activity. These features make PPC suitable for preparing medical packaging, dressings and other devices (Quan et al., 2021). In addition, PPC can be used as bone fixation materials, surgical sutures, and as “additives” to improve the properties of other polymers (Yang et al., 2014; Jing et al., 2015; Manavitehrani et al., 2017; Chen X. et al., 2022). Yang et al. applied PPC to improve the performance of PLA. The elastic composite of β-TCP/PPC/PLA showed non-toxic to osteoblast-like cells. The tensile strength of the composite material was close to 1MPa, and the elongation at break (%) was ∼1100%. The modified PLA-derived composite showed advanced mechanical and biological properties (Yang et al., 2014). In the field of dentistry, sodium fluoride-PPC (NaF-PPC) composite strips obtained by adding filler of NaF into melting PPC, exhibited excellent remineralization and antibacterial properties, and might be used for preventing dental caries (Chen X. et al., 2022). As the most promising CO_2_-based polymers that can be used in numerous biomedical applications, PPC and its derive nanocomposites merits further exploration.

### Poly (cyclohexene carbonate)

Based on ROCP of CO_2_ and cyclohexane oxide, poly (cyclohexene carbonate) (PCHC) is another kind of most common and well-studied CO_2_-based polymers. PCHC contains a six-member ring structure, which is more rigid and has better thermal performance than PPC (Welle et al., 2007; Liu et al., 2021). At room temperature, it is a brittle material and has high Tg (>100°C). It is a unique epoxide/CO_2_ copolymer that can be used at high temperature. The hydrophobic cyclohexane groups around the main chain of PCHC molecular make it more hydrophobic and less biodegradable. These factors make PCHC more difficult to be applied directly. However, composites of PCHC combined with other materials have been widely tested for application in drug-loaded nanoparticles, hydrogels, pore-forming substances, biodegradable surfactants, and regenerative scaffolds, etc (Liu et al., 2021). Alexander et al. prepared custom tissue stents of PCHC by electrostatic spinning technology (Welle et al., 2007). Zhang et al. used cationic PCHC as drug carriers to wrap therapeutic siRNAs. Serving as a siRNA carrier, PCHC showed good biodegradability, negligible cytotoxicity, and high transfection efficiency. It had been demonstrated that PCHC/siRNA nanocomposites had promising potential in pancreatic cancer treatment (Zhang X. et al., 2021).

### Poly (ethylene carbonate)

As a well-studied fatty acid polycarbonate, poly (ethylene carbonate) (PEC) obtained by ROCP of ethylene oxide and CO_2_, shows unique surface erosion caused degradation both *in vitro* and *in vivo*. As a result, drug release behavior of PEC-based delivery systems usually is directly correlated with polymer mass loss, which would improve the predictability of the drug release profile these drug delivery systems (Bohr et al., 2016). On the other hand, PEC degradation could be triggered by some special enzymes and cells. This feature would do good to realize the drug release at particular locations *in vivo*. Priemel et al. (2018) had found that adding PEC to PLA microparticles could increase rifampicin release and enhance antibacterial activity. Macrophage induced surface degradation of PEC was investigated *in vitro*. Degradation of PEC with molecular weight of 41 kDa was slower than that of PEC with molecular weight of 200 kDa, which was proved to be attributed to macrophage induced surface degradation. And this work indicated that on-demand drug delivery induced by macrophages can be achieved with PEC-based drug delivery systems (Chu et al., 2014). As PEC has a short degradation period, typically completed within 2–4 weeks, it is difficult for PEC to be used in long-term drug delivery (Bohr et al., 2016). By embedding anti-oxidants into the PEC polymer matrix to scavenge the free radicals produced by phagocytes, Selvakumar et al. (2016) had slowed down the degradation rate of PEC. This work revealed the degradation mechanism of PEC, and provided a theoretical basis for developing long-term drug delivery systems with controlled release behaviors.

### Poly (butyl carbonate)

Poly (butyl carbonate) (PBC), derived from monomers of epoxy butane and CO_2_, has good qualities of elasticity, tensile strength, elongation at break over 400%, Tg of −32°C. Despite of PBC’s good biocompatibility (Cai et al., 2017), its poor thermal stability and low melting temperature (∼55°C) have restrict its applications. Thus method of blending PBC with other polymers, has been frequently utilized to enhance the qualities of PBC-derived materials for a variety of applications (Wang et al., 2012; Wu et al., 2015; Li et al., 2019a). PBC has been transformed into biodegradable nanofiber membranes by numerous researchers for biomedical applications. For example, PBC/PLA/chitosan composite nanofiber membranes prepared by electrostatic spinning technique, had exhibited outstanding hydrophilicity, excellent antibacterial activity, and mechanical properties (Gu et al., 2019). In another work, axial electrostatic spinning was utilized to create core-shell PVA/PBC composite nano-fibers, which were then used as drug carriers of doxorubicin (DOX). The attachment and proliferate of SKOV3 ovarian cells could be inhibited by this DOX-loaded PVA/PBC core-shell nano-fibers, which indicated that as prepared nanocomposites have the potential to be used in tissue engineering and chemotherapy (Yan et al., 2016). Likewise, PBC can potentially be used as drug delivery vehicles for targeted therapies (Liu et al., 2007; Gu et al., 2018). Gu et al. (2018) prepared biodegradable nanofibers made of PBC, PLA, and graphene oxide (GO), and employed this PBC/PLA/GO composite nanofiber as a carrier for anti-tumor drugs. The findings indicated that the PBC/PLA/GO nanofiber matrix had the dual function of supporting cell imaging and drug delivery, which was promising for up-coming biomedical applications. PBC can also be added to other biodegradable polymers to enhance their mechanical and chemical characteristics. For instance, Wu et al. indicated that melt-blending PBC and PPC in an intermittent mixer might enhance PPC’s processing qualities and serve as a toughening agent (Wu et al., 2015). With good overall performance and low-cost, PBC is considered to be one of the most promising new biodegradable polycarbonates and deserves further development and utilization.

### Other CO_2_-based polymers

In addition to the common polycarbonates mentioned above, there are numerous o CO_2_-based polymers that have applications in the biomedical field. Poly (trimethylene carbonate) (PTMC) has excellent biocompatibility, biodegradability and flexibility, and is very attractive for applications as nanocarriers of pharmaceuticals. It had been found to have greater kinetic stability and enhanced drug transport, exhibiting excellent chemotherapeutic efficacy against various cancers (Wang et al., 2013; Jiang et al., 2014; Li et al., 2019b; Zhou et al., 2020; Zou et al., 2020). In addition, PTMC can be used as hydrogels or 3D printed scaffolds for tissue engineering, such as vascular grafts and bone regeneration scaffolds (Song et al., 2011; Schüller-Ravoo et al., 2013). In a recent study, fabricated custom scaffolds for orbital floor repair by stereolithography using 40wt% hydroxyapatite in PTMC resin, which promoted vascular neovascularization and bone morphogenesis in the orbital floor (Guillaume et al., 2020). Poly (limonene carbonate) (PLC), generated from limonene oxide and carbon dioxide, is then widely used in coatings and is a promising bio-based alternative (Li et al., 2017; Li et al., 2021). Poly (propylene carbonate maleate) (PPCMA) is a new kind of CO_2_-based polymers produced by ternary polymerization of PO, CO_2_, and maleic anhydride. PPCMA’s primary chain contains a double bond that gives it versatility and practicality, enabling its widespread application in numerous research domains. Liu et al. (2011) encapsulated PPCMA by microencapsulation and controlled drug methanesulfonic acid. The utility of PPCMA for controlled release of drug pazufloxacin mesylate revealed the potential of PPCMA as a long-term drug sustained release carrier. Additionally, absorbable CO_2_-based polymers can be developed as tracers to direct imaging agents to certain areas in applications of medical imaging, anti-cancer immunity therapy, antimicrobial materials, etc (Ding et al., 2012; Voo et al., 2015; Zou et al., 2016; Zou et al., 2017; Lee et al., 2020; Liang et al., 2021).

## Conclusion

In this review, focusing on the reduction of greenhouse gas emissions by biomedical polymer products, recent advances in studies on nanocomposites of CO_2_ fixation derived reproducible polymers have been reviewed. The CO_2_ fixation derived renewable polymers have been divided into three groups, namely naturally-derived polymers, synthetic renewable polymers and CO_2_-based polymers. As most of the CO_2_ fixation derived renewable polymers are biocompatible, non-cytotoxic, biodegradable and eco-friendly, there are more and more scientists engaged in researches on developing new kinds of biomedical materials by preparing nanocomposites of them. So far, great majority of research works in this field have been focused on high value-added biomedical applications, including tissue engineering, drug delivery, cancer treatment and organ transplantation, etc. However, the overall consumption of biomedical materials in these field is relatively small, which has a less important impact on reduction of CO_2_ emissions. In comparison, the researches on eco-friendly materials applied for disposable biomedical products, including diagnostic consumables, medical protective items (protective suits, face masks, goggles, surgical gloves, etc.), other medical consumables (sample tubes, drug packaging, medicine containers, syringe, infusion devices, etc.) and medical packaging materials, are obviously less than the former direction. Unfortunately, the raw materials of these low-cost disposable biomedical products, which have been ignored by scientists, are mainly petroleum-based polymers. Most of these consumable products have relatively short life cycle, and play a leading role in environmental pollution caused by biomedical polymer materials. Therefore, the authors suggest that more attentions and efforts should be given to develop new materials with small carbon footprint, based on CO_2_ fixation derived reproducible polymers for these short-life biomedical products in future.
